# Perceptions, practices and health seeking behaviour constrain JE/AES interventions in high endemic district of North India

**DOI:** 10.1186/s12889-017-4654-4

**Published:** 2017-08-08

**Authors:** Sanjay Chaturvedi, Neha Sharma, Manish Kakkar

**Affiliations:** 1 Department of Community Medicine, University College of Medical Sciences, New Delhi, 110095 India; 2 Public Health Foundation of India, Gurgaon, Haryana 122002 India

**Keywords:** Perception, Health seeking behaviour, Community, Japanese encephalitis, Uttar-pradesh, India

## Abstract

**Background:**

Acute Encephalitis Syndrome (AES) and Japanese Encephalitis (JE) stay as poorly understood phenomena in India. Multiple linkages to determinants such as poverty, socio-economic status, gender, environment, and population distribution, make it a greater developmental issue than just a zoonotic disease.

**Methods:**

A qualitative study was conducted to map knowledge, perceptions and practices of community and health systems level stakeholders. Seventeen interviews with utilizers of AES care, care givers from human and veterinary sectors, Non-governmental Organizations (NGOs), and pig owners and 4 Focused Group Discussions (FGDs) with farmers, community leaders, and students were conducted in an endemic north Indian district-Kushinagar.

**Results:**

Core themes that emerged were: JE/AES been perceived as a deadly disease, but not a major health problem; filthy conditions, filthy water and mosquitoes seen to be associated with JE/AES; pigs not seen as a source of infection; minimal role of government health workers in the first-contact care of acute Illness; no social or cultural resistance to JE vaccination or mosquito control; no gender-based discrimination in the care of acute Illness; and non-utilization of funds available with local self govt. Serious challenges and systematic failures in delivery of care during acute illness, which can critically inform the health systems, were also identified.

**Conclusion:**

There is an urgent need for promotive interventions to address lack of awareness about the drivers of JE/AES. Delivery of care during acute illness suffers with formidable challenges and systematic failures. A large portion of mortality can be prevented by early institution of rational management at primary and secondary level, and by avoiding wastage of time and resources for investigations and medications that are not actually required.

**Electronic supplementary material:**

The online version of this article (doi:10.1186/s12889-017-4654-4) contains supplementary material, which is available to authorized users.

## Background

Japanese encephalitis (JE), caused by a flavivirus transmitted by mosquitoes, is an outbreak prone zoonosis posing significant public health problem in some parts of India for several decades. The disease manifests as acute encephalitis syndrome (AES), and is associated with neurological complications, disability and mortality, especially among children. From the 1970s until around 2010, infection with JE virus (JEV) was considered to be the leading cause of AES in the JE belt of India, which includes Kushinagar District in the state of Uttar Pradesh (UP). However, several interventions including JE vaccination in endemic areas have failed to reduce the incidence of AES in children, including JE. As a result, there has been an ambiguity with regards to the epidemiology of AES in the JE belts of UP. Moreover, in recent times, the assertion that JEV is the leading cause of AES has been questioned, and other infectious agents, such as enteroviruses, have been reported as a cause of AES in UP and other parts of India [1–6].

Commonly referred as ‘*Mastishk Jwar*’ or ‘*Dimaghi Bhukhar*’ in endemic districts of UP, JE stays as a poorly understood phenomenon, both at social as well as at biomedical levels. In addition to the ecological factors (human, animal and environmental risk factors), there are societal factors that could explain the missing links in our understanding of JE and AES epidemiology. Social, cultural, agricultural and occupational practices, health-seeking behavior, gender roles, and human interaction with animals are all important factors that have failed to grab the attention of researchers and program managers and hence not suitably informed the intervention strategies for prevention and control of JE. Furthermore, the vast diversity of these ecological and social factors has led to different patterns of epidemiologic risks associated with JE incidence in different states and districts. Thus, JE is not merely a zoonotic disease, but a greater developmental issue with multiple linkages to several social and cultural drivers.

The JE-endemic district of Kushinagar in eastern Uttar Pradesh is a predominantly rural and agrarian district [7]. Nestling in the north east tip of the state. Part of the *‘Terai’* region (foot hill/low land), with a majority of the population practicing paddy cultivation, it is a densely populated area with high levels of poverty, unemployment, illiteracy and chronic undernutrition, combined with a near absence of basic facilities e.g. safe drinking water, electricity, sanitation and hygiene [8, 9]. Many rural dwellers live in close contact with animals, including pigs that are amplifying hosts of JE virus [10]. Man is an end line host for the JE virus and the natural life cycle involves pigs, ardeid birds and mosquito vector.

A qualitative enquiry was conducted to gain insights into societal and health system factors that influence JE transmission and disease outcomes. Perceptions of some key stakeholders (clients, service providers, and society) on awareness, causation, prevention and health seeking behavior related to JE/AES were mapped. Subsequent sections present and discuss the evidence generated through inductive methods.

## Methods

We used qualitative research methods to map the perceptions and practices of various stakeholders on JE/AES transmission, prevention, control and treatment during acute illness. The Institutional Ethics Committee, Public Health Foundation of India, reviewed and approved the study. Written informed consent was obtained from all the participants.

### Study setting

**Fig. 1:**
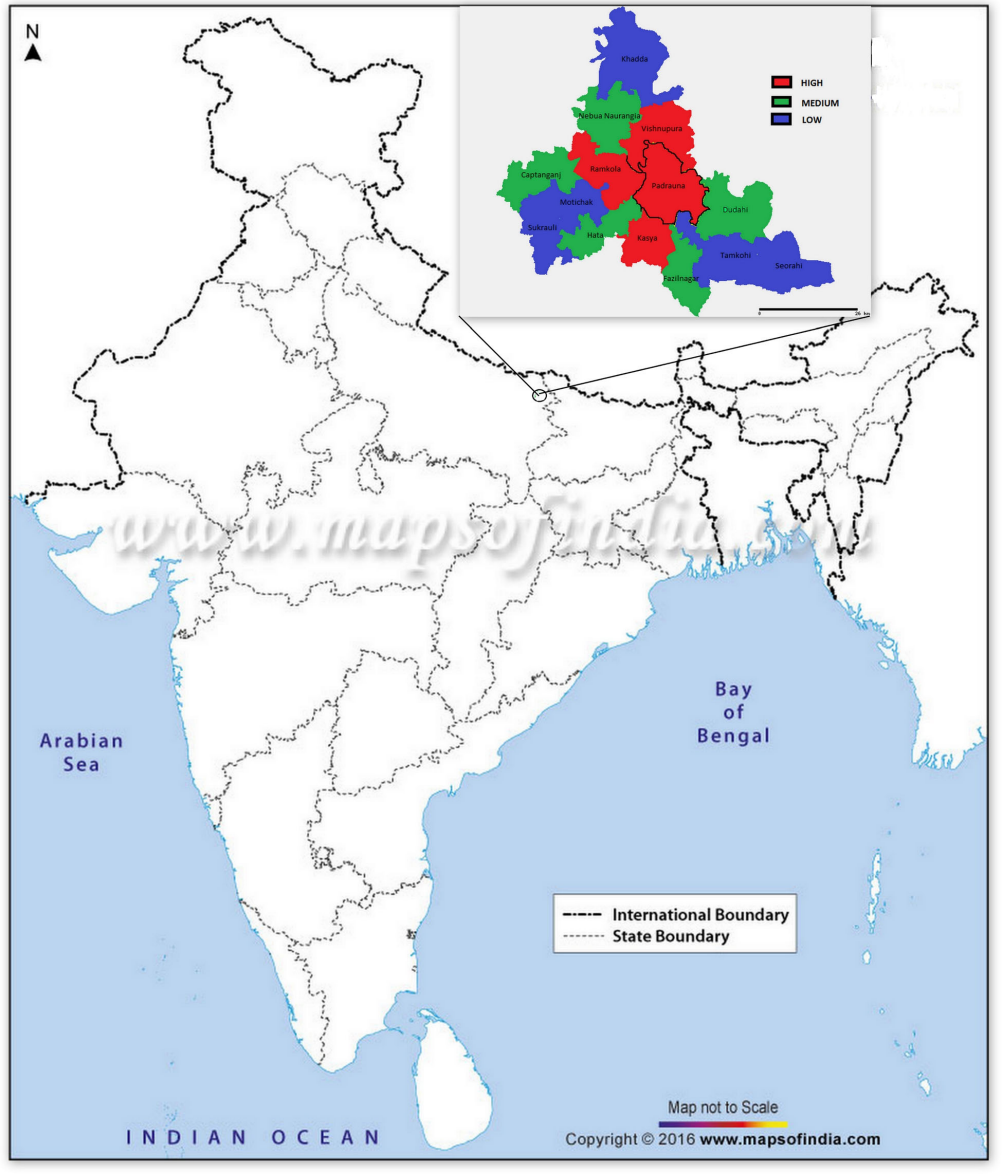
Map of Kushinagar. Block wise map of Kushinagar showing three blocks, Padrauna, Kaptanganj and Khadda, selected for the study on the basis of high, medium and low JE/AES disease burden. Permission: copyrights © mapsofindia.com and copyrights © National Informatics Center, Uttar Pradesh, Lucknow [33, 34]

Kushinagar, the district with highest JE burden in the state of UP, was selected as the study site. Kushinagar district is located in north east section of the state of UP and has reported high numbers of the total JE/AES cases in India. It is comprised of 14 administrative blocks, and three blocks, Padrauna, Kaptanganj and Khadda, were selected for the study on the basis of high, medium and low JE/AES disease burden (Fig. 1).

### Sampling

The sample included farmers, community leaders, pig owners, students, AES patient,; and medical and veterinary providers, Non-Governmental Organizations (NGOs), to obtain a holistic picture of JE related perceptions, practices and health seeking behaviour. Non probability, purposive sampling technique was used. JE cases were traced with the help of the list procured from the district health system.

### Data collection methods and instruments

Interviews and Group Discussions were used to collect qualitative data. An exploratory study assisted the research team to observe the locale, community perspectives, and real situations. Based on the outcome of several tool development workshops; core group meetings; and the information emerging from the exploratory study helped in developing the interview and group discussion questions. The domains of inquiry covered by interviews and group discussion instruments were as follows: significance of JE/AES as a health problem; causation of JE/AES; prevention and treatment of JE/AES; vaccination against JE; vector-control; care seeking during acute illness of JE/AES; and health systems’ response to JE/AES (Additional file 1). Profile of stakeholders interviewed and group discussions conducted in each of the selected blocks is provided in Tables 1 and 2.

**Table 1:** Profile of stakeholders for interviews and group discussions

Stakeholders	Padrauna	Kaptanganj	Khadda
Pig Owners	1	1	1
Utilizers of care of acute illness (AES)	2	2	2
Non-Utilizers of care of acute illness (AES)	Not found (during timeline of data collection)		
Representatives of Non-Government Organizations	1	1	1
Health care providers (Human)	-	ANM-1	PHC Doc-1
Health care providers (Veterinary)	-	LEO-1	Block VO-1
District Level Providers: Human Health-1 + Veterinary Health-1	1 + 1	NA	NA
Total IDIs	17		

a. *AES* Acute Encephalitis Syndrome, b. *ANM* Auxiliary Nurse Midwife, c. *PHC- Doc* Primary Health Center - Doctor

**Table 2:** Profile of stakeholders for group discussions

Stakeholders	Block	No. of group discussions
Farmers	Khadda	1
Community leaders	Kaptanganj	1
Students (11–15 years)	Padrauna	2
Total group discussions	4	

Interview and group discussion were recorded and handwritten verbatim notes were later supplemented by transcripts of audio tapes and then translated into English. All interactions were conducted in the local languages. The investigators were familiar with the local dialect, customs and culture. Interviews lasted for approximately 30–50 min, while group discussions ranged from 80 min to 2 h. Group discussions consisted of 7–12 participants from community.

### Data processing and analysis

Data were analyzed separately for each category of stakeholder and then compared to assess similarities and differences in perceptions across stakeholders. The triangulation was done at two levels – across methods and across respondents. The consistency indicating towards a substantive significance was also explored [11]. We adopted the Grounded Theory approach to develop an inductively derived explanation of data [12] instead of forcing or testing an “a priori” hypothesis [13]. The evidence and explanations were allowed to emerge through following stepwise process of analysis [14, 15]. Open coding (free listing/fracturing of responses, domain formation, assigning a domain code to each free listed response, and organizing the data); 2. Axial coding (relationship/connection between categories/sub-categories); 3. Selective coding (identifying core themes to which several other categories/sub-categories are related); 4. Reduction (getting the big picture); and 5. Allowing a model to emerge through constant comparison, which included the most important core themes.

### Emergence of proposed model

Concept identification began with the first set of interviews where data collection was alternated with analysis. Initially open coding was done by opening up the text of the interactions and subjecting them to intensive scrutiny. We also identified properties and dimensions of the phenomena, giving it specificity [14]. A point when data seemed repetitive, data saturation was achieved. Data were then woven around phenomena by axial coding. The major themes began to emerge from the data. Finally in selective coding, core themes emerged. The model finally arrived and went beyond mere reconstruction of events, it was a co-construction between researchers and participants [16]. It was also reflective of ground reality and the conditions that led to these problems [17].

### Quality assurance measures

A regular review by a multi-disciplinary team comprising of program evaluation experts, health social scientists, and public health specialists assured the quality of protocol and instrument development; data collection; analysis and interpretation. All group discussions were facilitated by senior investigators, and transcriptions and translations were cross checked for completion and accuracy.

## Results

Two emerging trends were noted in the perceptions of the respondents: a trend of synergy, and a trend of divergence of views, across stakeholders. Findings are discussed through the core themes on each of these two arms of synergy and divergence of perceptions that emerged inductively from the evidence generated from various stakeholders representing community, health care system, and non-government organizations. Quotes given with the core themes echo and represent the sentiment of majority/most of that sub-group of stakeholders.

### Core themes showing synergy across respondents

Themes discussed below are those that rose from similar voices and perceptions across stakeholders (especially, the clients and providers).

#### JE/AES is a deadly disease, but not a major health problem

JE/AES was not mentioned as a major public health problem by the community or health care providers. However it was considered a serious and deadly disease by all the stakeholders. JE was often linked to young children, high mortality and lifelong disability. Community stakeholders as well as human health care providers considered other diseases like diarrhea, fever, seizure, malnutrition, cough and cold to be more prevalent in the community. JE was neither amongst the top priority diseases nor did it figure amongst the most prevalent diseases. Diseases perceived to be most prevalent were: diarrhea, malaria, malnutrition, typhoid, and jaundice.

#### JE/AES was associated with general unhygienic conditions with no clear link with pigs

JE/AES was often associated with general filthy conditions, dirty water and mosquitoes. Both, the community and the provider side stakeholders interlinked the three factors mentioned above but the linkage with mosquitoes was not so strong as it was explicitly shown with overall filthy conditions that generated mosquito menace as well. Often, the filth and dirty water was perceived to be causing, besides causing several other diseases, and mosquitoes were seen as an added nuisance generating out of dirty water. The phrase ‘*filthy conditions’* was frequently used to describe the physical condition of the area and one of the main factors held responsible for the disease. Dirty water had many versions, like, stagnant/accumulated water, open sewage drains and unsafe water used for drinking and other purposes.

*“She (the child) use to play in the dirt and that can be one of the reasons why she got JE/AES”. (Interview, Mother, Padrauna).*

Pigs were not mentioned by the respondents until probed. When respondents were asked regarding any animal in particular being responsible for the disease, pigs were mentioned, especially in the pig owning villages. Pigs were not associated with JE directly but with filth and unhygienic surroundings they lived in.

*“Pigs stay in unhygienic conditions, which results in various diseases” (Focus Group Discussion, Farmers, Khadda).*

However people also believed that pigs helped in maintaining cleanliness in the village, as they ate dirt along their way. No clear linkage of pigs with JE was established, pigs were considered as one of the factors, amongst many others, which might be responsible for JE/AES.

#### Pig owners’ perception

Pigs were not commonly found in every village of the block. There were certain villages that had few families owning them. These families predominantly belonged to a marginalized caste ‘*Dom*’. The *Doms* tend to live in the southern part of the villages and were uniquely placed in the villagers’ inter-community structure. Physically, they were living on the margins and were considered of low caste community. However, they served as important actors in special occasions such as marriages and funerals in the village. For pig owners, pigs were the major source of livelihood option they had. A common phenomenon noticed around the houses of pig owners was the presence of bamboo. One possible reason behind this could be that along with selling of pig meat they also made products out of bamboo e.g. baskets etc. Pig owners felt that pigs did not play a significant role in the transmission of JE. They put across their case by emphasizing on the fact that if pigs were responsible for causing JE then their children would have been among the first ones to be infected.

*“Pigs do not play any role in diseaese tramission, had it been the case we would have been caught by diseases as we live in close proximity with them”. (Interview, Pig Owner, Kaptanganj).*

**Fig. 2:**
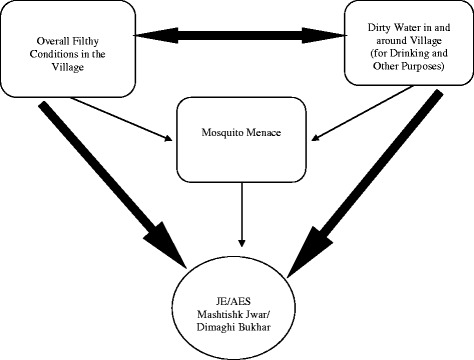
Perception of factors related to Japanese encephalitis/acute encephalitis syndrome in non-pig owning villages. The perception of factors related to Japanese encephalitis/acute encephalitis syndrome in non-pig owning villages. People linked overall filthy conditions and dirty water around the village as main reasons for JE/AES. Pigs were not mentioned in the cycle at all. *Thick arrows* represents strong association

**Fig. 3:**
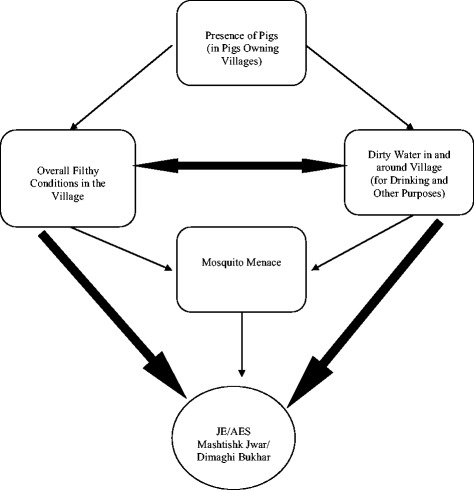
Perception of factors related to Japanese Encephalitis/Acute Encephalitis Syndrome in Pig Owning Villages. The perception of factors related to Japanese encephalitis/acute encephalitis syndrome in pig owning villages. Respondents did not draw any strong associations of pigs with reference to JE, however pigs were mentioned with filth and unhygienic surroundings they lived in. *Thick arrows* represents strong association

Interestingly over the years their families did not have any cases of JE/AES, a version that was echoed and corroborated by other villagers. However, they expressed that if pigs were harmful, they would give up pig ownership, but urged the government to provide them with alternative employment options to sustain their families. Figures 2 and 3 illustrate the perceptions about the relationship within these factors, and with JE/AES as the outcome, in pig owning and non-pig owning villages.

#### Minimal role of accredited social health activist (ASHA)/auxiliary nurse midwife (ANM), government health workers, in the first contact care of acute illness: First contact care is provided by non-formal prescriber in most cases

None of the community members mentioned about the role of the peripheral health workers in cases of acute illness care.

*“ASHA, only handles deliveries in the village. She just takes the women to the hospital. ASHA and Anganwadi workers are only for name sake” (Focus Group Discussion, Community Leaders, Kaptanganj).*

As the child developed fever, the first contact point was a non-formal prescriber in most cases and patients were frequently taken to ASHA/ANM (sub centre) in the village. Once a JE/AES case was detected there was no involvement of village level health care workers. However some patients recalled that after the child died, peripheral level health functionaries visited their household to inquire about the child. Often it was pointed out that the roles of peripheral health care workers were restricted in aiding deliveries and giving vaccinations (polio drops were commonly mentioned). In some villages respondents also mentioned irregular field visits of the ASHA/ANM.

#### No social or cultural resistance to JE vaccination or mosquito control activities

In the midst of conflicting perceptions between clients and providers about the JE vaccine coverage, one point reflected synergy. There was no social or cultural resistance observed or reported against JE vaccination. Not even a single respondent pointed out any social or cultural resistance towards the vaccine or other mosquito control activities e.g. fogging in the area.

#### No gender-based discrimination in the care of acute illness

Respondents from both sides, community as well as health care system, expressed gender based discrimination was not practiced or seen as far as treatment seeking behavior of clients. Respondents did not differentiate between male/female children when it came to treatment. They emphasized on that children of both sexes were given equal treatment in terms of promptness, choice of service delivery and other important parameters affecting their health.

#### Non-utilization of funds available with village Pradhan (local self government)

The responsibility for implementation of activities of Village Health and Sanitation Committee (VHSC), envisaged under the National Rural Health Mission (NRHM), rests with local self-government/*Panchayat Raj* Institutions (PRIs). The committee is entitled to utilize an annual grant of Rs.10, 000/− as a village health fund which is a community resource. Nutrition, education & sanitation, environmental protection, public health measures are the key areas where funds may be utilized. The respondents were well aware of funds being available with Village *Pradhan* (local self-government functionary), but repeatedly mentioned about the underutilization/non utilization of funds. The perception of most of the respondents was that these funds were seldom utilized for any sanitation or mosquito control measures like fogging and spraying. Spraying and fogging took place about 2 years ago. No one recalled any recent spraying or fogging made by *Pradhans* in this regard, but did recall these activities about 2 years ago.

### Core themes showing divergence across respondents

Themes discussed below are those that showed a divergence of perceptions across stakeholders (especially, the clients and providers).

#### Awareness about JE/AES

Community’s perspective: The community level respondents expressed about a low awareness with regard to the disease. Most respondents were not aware of the causative factors, treatment, prevention and control of diseases. Mothers of children who had *JE/AES* were unsure about many aspects of the disease. Respondents also expressed a lack of awareness activity being carried out in the community by any agency. Community leaders pointed out that because of lack of awareness, the community is not able to prevent the disease or save the patient from serious consequences of the disease.

*“I don’t know how my child got JE/AES, he died suddenly (child). Doctors will be able to tell the reasons. I have heard it is because of mosquito bite and cold” (Interview, Mother, Khadda).*

Providers’ perspective: Health care providers believed that there is a good amount of awareness in the community. According to them regular awareness activities, awareness among Village *Pradhans* and other community leaders has led to a higher acceptance of preventive measures and early reporting of cases.

*“People are not scared of the disease, as they were earlier. Now people are informed and want to get their children vaccinated” (Interview, PHC, MO, Khaddha).*

#### Incidence of JE/AES

Community’s perspective: Respondents typically mentioned an increase in number of cases over the years.

*“Incidence of JE/AES has increased in the last 5 years” (Focus Group Discussion, Community Leaders, Kaptanganj).*

People cited examples from villages and surrounding areas of JE/AES cases they were aware off. They heard about the cases through word of mouth, media or saw them in the hospitals.

Providers’ perspective: Peripheral health workers as well as the Medical Officer in charge – Primary Health Centre (Mo/Ic-PHC) mentioned that a regular vaccination programme and awareness in the community has led to a declining trend in the incidence of disease during the last 5 years. Most of the provider side respondents held a perception that the disease was under control in the area.

*“Cases of JE have reduced, morbidity has reduced” (Interview, PHC, MO, Khaddha).*

#### JE vaccination

Community perspective: Community level respondents could hardly recall any JE vaccination activity between March 2011 (*Holi* festival) and December 2012. In some villages very few children were reported to be vaccinated in December 2012 on an ad-hoc basis. The description of the vaccination activity reported by the community was neither fitting the routine immunization nor a door-to-door campaign. People often compared JE vaccine with Supplementary Immunisation Activities (SIA) against polio. The comparison was primarily done in terms of better awareness activities, social mobilization and implementation of the polio immunisation rounds. Respondents repeatedly expressed that no awareness in regard to the JE vaccine had been made by the government. They believed that vaccine is stocked and is not made available to the people. The discrimination practiced by health workers in giving routine immunisation, in general, was also articulated by the community. A perceived preference was given to children of affluent and influential families of the village. It was also reported that some health workers charged money (Around Rs. 10–15 per child) for giving routine immunisation, which is a free service.

*“Vaccination only reaches VIPs. ANM charges Rs. 10 per vaccine. We give her money but definitely get our children vaccinated” (Focus Group Discussion, Community Leaders, Kaptanganj).*

Providers’ perspective*:* Providers presented a very different view from that of the community. According to them, JE vaccine (JEV) rounds were implemented on a regular basis. Health workers collected the vaccine from the primary health centre based on the need assessment in the villages and administered to most of the target children. They also mentioned that vaccine was given through routine immunisation as well as through supplementary immunization activities. Providers also believed that because of regular vaccination rounds, JE incidence had reduced over the years.

*“The vaccine is given through routine immunization. It is given twice a week. The no. of cases have reduced because of JE vaccine. Initially people use to resist the vaccine, but now people get their children” (Interview, PHC, MO, Khadda).*

#### Care of acute illness in health care system

Community’s perspective: Most of the community side respondents were visibly dissatisfied with the health care provided by the government. Some major points of discontent were as follows:

- Government health centers and hospitals were perceived to be filthier than their village surroundings and no sanitation was maintained. Quite frequently 2 patients were to occupy one bed. Basic needs like potable water, food and medicines were not available most of the time.
- Respondents mentioned that once they would enter the government health system, they were asked for money at each step from admission to outcome.

*“I have been to a PHC twice. Government functionaries ask for money” (Student, Focus Group Discussion, Padrauna).*

They believed that the system is actually run by touts who demand money even for services that are available for free. Unnecessary diagnostic tests and medications are forced on them.

- They frequently expressed that a majority of the health care workers and doctors in the government hospitals and health centers behave in a rude and insensitive manner.
- There was a strong perception about discrimination among patients on basis of their socio-political connections and paying capacity.

*“Doctors prescribe us medicine and we have to buy it. If the patient has money the doctor pays attention, otherwise they don’t” (Farmer, Focus Group Discussion, Khadda).*

Respondents had a similar perception about private clinics and nursing homes. However they found that the behavior of workers and doctors were better there. The cost in the private clinics was prohibitive, leaving most of the parents in an unfortunate dilemma. After spending much of their money at a private facility, they were either referred to –or- left with no choice other than to visit a government hospital, and once they entered the government system they faced what they perceived as exploitation. Discrimination based on socio-political connections and paying capacity was perceived to be comparable in government and private systems.

Providers’ perspective: Provider side respondents had a different perception. They felt that people wasted a lot of time before they visited a formal health functionary, resulting into delayed initiation of management, avoidable complications and mortality. Provider side stakeholders also expressed that they prefer to retain the patients at health centre or district hospital levels, depending on the seriousness of illness, but most of the time, parents and relatives force the doctors to refer the patients to a medical college. This happens inspite of counseling. They also expressed that the parents and relatives put a lot of pressure on the doctors to go for diagnostic tests and medications that are not actually required.

*“If by chance we are unable to save the patient, a lot of tension is created, attendants blame the doctor. They raise questions on our treatment and blame us for being careless and not referring the child to Gorakhpur” (Interview, PHC, MO, Khaddha).*

**Fig. 4:**
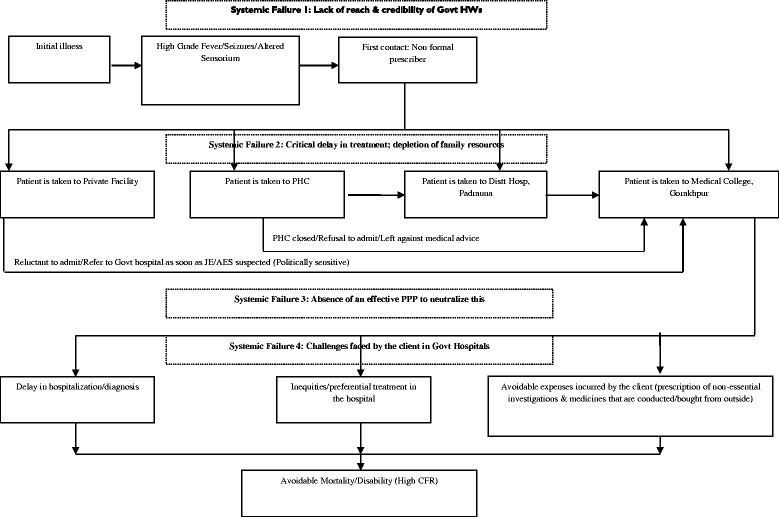
Care of Acute Illness: Emerging Pattern of Specimen Case Experience. Process of care of JE/AES patients based on an emerging pattern of specimen case experience and draws attention on challenges faced by people and some of the existing systematic failures

Whatever the critical forces, push or pull, the emerging narrative of a specimen case of acute illness, from the first contact with a non-formal prescriber to hospitalization in medical college, is constructed in Fig. 4 which also illustrates four levels of systemic failure in the care of acute sickness.

#### Training

##### Human health functionaries

District level medical officer reported to have attended a training program on clinical presentation, diagnosis, management of cases and prevention of JE. He asserted that similar training programs were conducted for peripheral health functionaries as well. However, he expressed a lack of coordination between human and animal health sectors. The peripheral health functionaries, on the other hand, could not recall any such training program or information-sharing being organized for them.

##### Veterinary health functionaries

The district level veterinary officer said that special trainings on JE was organized for all the veterinary doctors, livestock extension officers (LEOs) and paravets at all levels, but as seen in human health, block veterinary officer and livestock extension officer could not recall any training program being offered or information-sharing being organized for them or their colleagues, even within their own sector.

#### Participation of NGOs

Representatives of the NGOs underscored their participation in JE/AES control activities, especially in awareness campaigns, community mobilisation for JE vaccination and facilitating care during acute illness, but this was not corroborated by any respondent from any other stakeholder category.According to other stakeholders, including community members and health care functionaries, they had never come across any NGO activity in JE control. Nor were they aware of any NGO working in the field of JE.

## Discussion

The most insightful core themes emerging from these findings were: non-recognition of JE/AES as a major public health problem; lack of awareness of risk factors and causation; low coverage and ad-hoc management of JE vaccination activities; and some serious systemic failures in the care of acute illness. Lack of awareness across respondents, including provider side stakeholders, was worrisome. Even health workers and doctors were found wanting on the score of risk factors and causation of JE/AES. In this scenario, improving an abysmal state of information education and communication needs to be given priority before going for behavior changes in communication. Respondents, across categories, did not show any strong association between JE/AES and mosquito bites. With the available evidence, it is difficult to answer that these perceptions were shaped by ignorance –or- confusion, and to what extent. This may partially be a result of conflicting information about the etiology of AES in academic as well as public space. Lately, reports of AES being caused by water borne pathogens (especially enterovirus), insecticides used for *Litchi* fruit crop, and *Rickettsia prowazekii* transmitted by human lice have also reached popular media [1–6, 18, 19].

Some proportion of AES may actually be attributed to these causations but the presence of JE virus, vector, a large number of unimmunized children and absence of vector control activities makes it difficult to believe that JE ceases to be a major cause of AES in the area. The protection of humans against mosquito bite will continue to be a cardinal arm of intervention against AES, especially when the vector control is relatively difficult to achieve. Related evidence from malaria control activities in UP has indicated that most of the respondents were satisfied about the performance of long lasting insecticide treated nets (LLINs) [20]. Along with IEC efforts, social marketing of LLINs at a subsidized price or free supply to under-privileged sections during harvesting season could encourage people to buy and use LLINs [21]. Researchers from western India have reported that about 79% of the respondents were willing to buy treated nets [22].

Besides protection from mosquito bite, evidence emerging from anti-malaria program would also inform several other JE control activities. Recent qualitative investigations on barriers to malaria control among marginalized communities have suggested using culturally-appropriate information and education tools; making non-formal prescribers partners in malaria control; promoting within-village rapid diagnosis and treatment; increasing treated nets distribution and promoting their use as potential preventive strategies [23]. Barring the promotion of within-village rapid diagnosis and treatment, all other recommendations have a bearing on JE/AES control activities as well. However, the partnership with non-formal prescribers in JE control will have to be managed carefully. Such partnership should grow more in the area of primary prevention, case detection and surveillance. Considering closer links and networking with the private sector, an increasing role in case management may result in higher number of referrals to private clinics and prolonging delays in the initiation of rational case management.

Low reach and credibility of government health volunteers/workers (Accredited Social Health Activists/Anganwadi workers/Health Workers-Female) for seeking first-contact care during acute illness was another finding that demands serious attention, for this phenomenon sets in motion a chain of events leading to unfavorable outcomes. Going by specimen narrative, a non-formal prescriber was the first contact care-giver who usually referred the case to a private clinic. At the private clinic, the patient was treated for few hours to a couple of days before being referred to medical college. By the time patient reached medical college, much of the patient’s family resources were already depleted. Rational management of case was still on a weak ground even at the medical college level where several unnecessary diagnostic tests and medications were prescribed which had to be procured from outside. The first link in this chain was the first contact care-giver who was a non-formal prescriber in most cases. This has been corroborated by malaria related researchers who found respondents expressing a lack of trust in government health workers. This drove them to care-seeking from traditional healers and unqualified providers. Faith in the service provider and perceived effectiveness of available services were among major determinants of health care [24]. Other workers studying socio-economic, political, and cultural aspects in the implementation of the malaria control programme in south India have observed that it is ultimately the community that takes the major decision directly or indirectly and the health authority must guide them in right direction. They also reported that financial difficulties and meager budget allocation led to indifferent attitudes of *Panchayat* members towards health care [25]. On the other hand, findings in our study indicate that there was a strong perception across stakeholders about non-utilization of funds available with Village *Pradhans* for health related activities.

A large portion of mortality and complications can be prevented by: early institution of rational management at the primary and secondary levels; avoiding unnecessary referrals to medical colleges; minimizing discrimination based on social and political connections; and avoiding wastage of time and resources for investigations and medicines that are not actually required. Two common reasons of referral from a private care facilities were low paying capacity and political sensitivity associated with JE/AES case. Technically speaking, many of them may not actually be referrals but getting rid of a potentially difficult situation. Strengthening public-private partnerships at the local level may help in addressing these problems. Building a culture of rational use of diagnostic tools and medicines should never be an issue in educational institutions e.g. medical college but the social reality of health care is full of complex riddles and paradoxes. An encouraging finding in this study was the absence of discrimination between sexes in health seeking treatment seeking and other JE/AES related behavior, although malaria researchers from south have reported strong gender discrimination [25].

The presence of pigs around human dwellings was not identified as a major risk. Pig owners did not perceive them as contributing to JE threat. In fact, pig owners’ concluded that their children did not suffer with JE/AES, and this conclusion was echoed by other. At this stage and with this qualitative data, it would be premature to take a call on probable zoo-prophylaxis (protection posed by presence of pigs) instead of zoo-potentiation in this case. Nonetheless, similar perceptions among pig raisers in Bangladesh have been documented, although in a different context and with a little different meaning. Bangladesh pig farmers believed that disease causing pathogens in pigs could be transmitted from a pig to pig but not to humans [26]. Marginalization, stigmatization, poverty, and low levels of education among pig raisers in this region make it difficult to implement risk reduction measures e.g. vaccine of swine. Economic benefits might interest pig raisers in accepting interventions against pig-borne zoonoses. An active component of social and vocational support is required to reduce stigma and to demonstrate economic benefits of disease control [27].

Lately, there have been attempts to augment JE vaccination coverage and integrate this with routine immunization activities but unless we have an external evaluation of vaccine coverage there are no reasons to believe that the community perception on JE vaccination (between March 2011 and December 2012) documented in this study was way off the mark. The divergence in perception of client side and provider side stakeholders on JE vaccination was very wide. Ground situation may be improving now but we still need evidence generated from independent sources to support this. The data that fit this requirement are far and few [28]. Routine immunization (RI) are a good move but this would not be enough when the coverage of RI in the area is very low [29]. Supplementary Immunization Activities (SIAs) against JE will have to be systematically organized, and strengthened with better, equity-based micro-planning. Lessons learned from polio need to be factored here. It was remarkable to note that the cultural or social resistance witnessed against polio-SIAs in many areas of UP was not observed against JE vaccination. In fact, community level stakeholders were demanding JE vaccination campaigns [30]. However, it would be early to conclude anything at this stage since the JE-SIAs are yet to reach that phase of saturation and social fatigue.

The importance of breaking the silos can never be overemphasized in disease control, especially when we are dealing with a zoonosis. Not only was there a perceived lack of coordination between human and animal health sectors, peripheral functionaries in both sectors also expressed a lack of systematic flow of information within the sectors. Zoonoses do not respect such silos and frequently cross animal-human territories. Organizational and jurisdictional divides that exist between human and animal health agencies seriously impede cogent responses to zoonotic diseases. Some medical personnel have observed that agencies responsible for livestock and wildlife follow stand-alone and sometimes competing agendas [31]. To create a culture of systematically building the inter-disciplinary and inter-organizational bridges, some international efforts and evidence-based approaches like Eco-Health and One Health are now seeking global attention [32].

Lastly, at the level of academic culture, we need to address a riddle that haunts the program leadership caught between etiological enquiries and mitigation arm of action. Certain risk communications as well as etiological debates surrounding AES have filled the public space with conflicting information leaving many stakeholders confused [1–6, 18, 19].

## Conclusion

Placing undue attention on discovery-centric research may unfold as a costly mistake for a low or middle income country, when most of the deaths and disability can be averted by syndromic identification of case (AES) and early initiation of syndromic management. Sometimes, an elitist approach of etiological research may turn to be a liability on the program and people.

## Additional file

Additional file 1: Interview Guides. (DOCX 15 kb)
