# Supplementary material for: Perceptions, practices and health seeking behaviour constrain JE/AES interventions in high endemic district of North India
Source: BMC Public Health. 2017 Aug 8;17:645. doi: 10.1186/s12889-017-4654-4 (PMC5549343; doi:10.1186/s12889-017-4654-4)
Supplement: Additional file 1: — Interview Guides. (DOCX 15 kb) [file 12889_2017_4654_MOESM1_ESM.docx]

**Theme Guide**

**In depth Interviews**

| **Stakeholder** | **Themes** |
| --- | --- |
| **Pig Owners** | - Background details (related to pig ownership) - Problem of Mosquito - Dimaghi Bukhar Specific Perceptions - Child’s background (child that got Dimaghi Bukhar + other children in household) - Dimaghi Bukhar Case Study - Describe what happened to your child - Describe your experience from the point that you decided to seek help - Describe your experience with the health care giver - Impact of Dimaghi Bukhar - Retrospection |
| **Non Utilizers of Acute Health care** | - Mosquito problem - Dimaghi Bukhar Specific Perceptions - Children’s background (child that got Dimaghi Bukhar+ other children in household) - Dimaghi Bukhar Case Study - Describe what happened to your child - Describe your experience from the point that you decided to seek help from other sources - Describe your experience with this care giver - Impact of Dimaghi Bukhar - Retrospection |
| **NGO/CBO** | - Dimaghi Bukhar (occurrence, distribution, awareness, practices, issue worth intervening, perception of community) - NGO/ CBO Intervention - Retrospection |
| **Health Providers (ANM, PHC & CHC)** | - Disease Burden and AES - Dimagi Bukhar Epidemiology and Pathology - Interventions/Vector Control Measures - Vaccination - Treatment/Testing/Diagnosis |
| **District Health Providers** | - Disease Burden and AES - AES Epidemiology and Pathology - Interventions/Vector Control Measures - Vaccination |
| **Veterinary Health** | - Disease Burden and AES - AES Epidemiology and Pathology - Animal Rearing and Health - Interventions/Vector Control Measures - Vaccination |
| **Veterinary- District Level** | - Disease Burden and AES - Animal Rearing and Health - Vaccination |

**Focus Group Discussions**

| **Stakeholder** | **Themes** |
| --- | --- |
| **Students** | - Perceptions and Awareness about Diseases and Dimaghi Bukhar - Cause and transmission of Dimaghi Bukhar - Practices about Dimaghi Bukhar Prevention and Treatment - Vaccination - Vector-Control - Perceptions on Health System |
| **Farmer** | - Perceptions and Awareness about Diseases and Dimaghi Bukhar - Dimaghi Bukhar (general awareness, causative factors, occurance) - Cause and transmission of Dimaghi Bukhar - Perceptions, Awareness and Practices about Dimaghi Bukhar Prevention and Treatment - Vaccination - Vector-Control |
| **Community Leaders** | - Perceptions and Awareness about Diseases and Dimaghi Bukhar - Dimaghi Bukhar (occurrence, distribution, awareness, practices, issue worth intervening, perception of community) - Cause and transmission of Dimaghi Bukhar - Perceptions, Awareness and Practices about Dimaghi Bukhar Prevention and Treatment - Vaccination - Vector-Control - Perceptions on Health System |
